# Comparison of Regression and Classification Models for User-Independent and Personal Stress Detection

**DOI:** 10.3390/s20164402

**Published:** 2020-08-07

**Authors:** Pekka Siirtola, Juha Röning

**Affiliations:** Biomimetics and Intelligent Systems Group, University of Oulu, P.O. BOX 4500, FI-90014 Oulu, Finland; juha.roning@oulu.fi

**Keywords:** stress detection, wearable sensors, regression, classification

## Abstract

In this article, regression and classification models are compared for stress detection. Both personal and user-independent models are experimented. The article is based on publicly open dataset called AffectiveROAD, which contains data gathered using Empatica E4 sensor and unlike most of the other stress detection datasets, it contains continuous target variables. The used classification model is Random Forest and the regression model is Bagged tree based ensemble. Based on experiments, regression models outperform classification models, when classifying observations as stressed or not-stressed. The best user-independent results are obtained using a combination of blood volume pulse and skin temperature features, and using these the average balanced accuracy was 74.1% with classification model and 82.3% using regression model. In addition, regression models can be used to estimate the level of the stress. Moreover, the results based on models trained using personal data are not encouraging showing that biosignals have a lot of variation not only between the study subjects but also between the session gathered from the same person. On the other hand, it is shown that with subject-wise feature selection for user-independent model, it is possible to improve recognition models more than by using personal training data to build personal models. In fact, it is shown that with subject-wise feature selection, the average detection rate can be improved as much as 4%-units, and it is especially useful to reduce the variance in the recognition rates between the study subjects.

## 1. Introduction and Related Work

Wearable sensors are commonly used to monitor human motion based on inertial sensors such as accelerometer, gyroscope, and magnetometer. However, wearables can also include sensors to measure biosignals. In fact, nowadays wrist-worn wearable devices can include a wide range of biosensors, including photoplethysmography to measure blood volume pulse, thermometer to measure body temperature, and electrodermal activity sensor to measure galvanic skin response. Based on these, it is possible not only to monitor human motion, but they also enable a possibility to monitor other aspects of human behaviour and things happening inside human body.

Recently, there has been a lot of attention to stress and affect recognition using wearable sensors. For instance, in [1] four affect states (normal, happy, sad, fear, and anger) were recognized based galvanic skin response and blood volume pulse signals, and in [2] eight affect states were detected based on acceleration, electrocardiogram, blood volume pulse, and body temperature signals.

One especially interesting affect state to recognize is stress. There is evidence that well-being at work and working efficiency are connected [3]. This is because employees that are feeling well at work have better engagement, are more motivated and take fewer sick days. One of the factors leading to reduced work well-being and working efficiency is stress. To increase productivity, causes of stress at work should be studied, and before this can be done, methods to measure stress needs to be studied. Moreover, chronic long-term stress can impact on brain structures involved in cognition and mental health [4] and cause many other problems as well for instance by effecting to the immune system [5].

Stress detection based on wearable sensors have been studied a lot, probably more than detection of any other affect state. For instance, in [6] a classifier to detect high and low stress, as well as, non-stressful situations at laboratory conditions based on wearable wrist-work sensors. The result of the paper was these two classes can be detected with the accuracy of 83%. Moreover, in [7] a binary classifier to stress and non-stressed state was trained, and it was noted that stress can be detected using sensors of commercial smartwatches. There are also several other studies showing that stress detection based on classification models can be performed with high accuracy using user-independent models (see for instance [8,9]). However, according to some studies, the accuracy can be further improved by using models based on personal data ([10,11]).

What is noticeable is that most of the stress and affect detection studies are based on classification methods commonly used in human activity recognition, for instance [6,12]. These methods include feature extraction using sliding window technique and usage of the same classifiers. The reason for this is probably that the devices used in both studies are similar, or at least partly similar, as they both are using wearable devices and the data obtained by the sensors of these devices. While the recognition accuracies shown in stress detection articles are good, the problem is that they are based on classification of discrete target variables. However, stress and other emotions are not discrete phenomenons as for instance the level of the stress of a person can be high or low or anything between these. In [6], it was suggested that discrete classification results could be transformed as continuous based on posterior values. Thus, still also in this case, the original classification to stress/non-stressed would be based on discrete target values. In fact, one problem is that in affect recognition studies it is already decided in the data gathering phase simplify the studied phenomenon by transforming a continuous phenomenon as discrete due to difficulty to gather continuous target values. Of course, transforming continuous phenomenon as discrete is far from optimal solution, and this type of problem simplification can cause problems in the modelling phase. In fact, this means that a problem that originally was regression problem is transformed as classification problem. Therefore, a better option would be to base stress and affect detection to continuous target values.

While the most of the stress detection studies are based on discrete target values, there are some attempts to gather and analyze continuous target values for stress detection. In [13], stress while driving a car was studied. In the study, continuous target variables for stress level were created based on video recorded to analyze driver’s facial expression, body motion and road conditions. Another study, where continuous target values for stress detection were gathered is [14,15]. Furthermore, in [14] the study concentrates on analyzing stress while driving, and during the data gathering, driver constantly estimated his/her own stress level. However, while in these two datasets the data contains continuous target values, in both cases the final analysis is based on discrete targets created based on continuous targets. On the other hand, there is also studies where stress and other affective states have analyzed using regression model (see for instance [16,17]; however, these are rare compared to studied based on classification methods.

As stress is not discrete phenomenon, it should be analyzed based on continuous target values. This means that instead of classification, the data should be analyzed using regression methods. This article is the first one where regression and classification models for stress detection are properly compared to experiment regression methods really out perform classification method. In addition, the cons and pros of classification and regression models are discussed. Moreover, user-independent and personal recognition models are compared. The study is based on a publicly open dataset making the reproduction of the presented results easy.

The article is organized as follows: Section 2 introduces the used dataset and Section 3 introduces the methods used in the experiments. Experimental setup and results are explained in Section 4, and finally, the discussion and conclusions are in Section 5.

## 2. Experimental Dataset

This study is based on publicly open data set called AffectiveROAD [14]. It contains data from nine participants (from now on these participants are called NM, RY, BK, MT, EK, KGS, AD, GM and SJ) measured using Empatica E4 wrist-worn device [18]. However, three participants performed data gathering session more than one’s, and therefore, the dataset contains data from 13 data gathering sessions. Participants wore this device on both wrists. E4 includes accelerometers (ACC), as well as, sensors to measure skin temperature (ST), electrodermal activity (EDA), blood volume pulse (BVP), heart rate (HR), and heart rate variability (HRV). In addition to E4 data, AffectiveROAD includes data from Zephyr Bioharness 3.0 chest belt. However, in this study, only Empatica E4 data are used as the focus of this article is in wrist-worn sensors. Moreover, to be able to better compare the results of the study to the results of previous studies, only data from right wrist-worn Empatica was used.

In the data gathering session, the task of the study subject was to drive car in a normal daily traffic. However, the session started with a rest period where study subject was sitting and resting in a car, eyes closed and engine running. This can be considered as baseline data. The actual driving consisted of driving at two types of roads: city driving and driving at highway. City driving is assumed to be stressful as it contains traffic lights, a lot of vehicles, pedestrians and bikes. On the other hand, a highway is a smooth road and driving there is assumed to be less stressful.

What makes this dataset special and unique is that it includes continuous subjective stress estimates which were collected by the experimenter sitting at rear seat while study subject was driving. Moreover, the driver validated these estimates after driving session. The scale for estimates was from 0 (=no stress) to 1 (=maximum stress). However, there are no stress estimates available from baseline. In this study, it is assumed that during the whole baseline session, the level of stress is 0. As this dataset contains continuous target variables, these data can be used for regression analysis as well as for classification after discretizing target values. More detailed information about the dataset can be found from [14].

For the model training signals from the data gathering sessions were divided into windows, and from these windows, features were extracted. Window size of 60 s was used in the experiment, which is the same as used in [7,19] for stress detection. The used slide was between two adjacent windows was 0.5 s. The features extracted from these windows were the same as the one’s used in [19]. Therefore, four types of features were extracted: features from the ACC signal, EDA signal, BVP signal and ST signal. Altogether 119 features were extracted.

## 3. Methods

Classification was is in this study based on Random Forest, as it was found as the best classification algorithm for stress detection in our previous study [7]. However, to found the most appropriate regression model for the purpose, several regression models were compared. The comparison was made using Matlab’s (version 2018b) Regression Learner application which enables fast experimenting with multiple regression algorithms. Regression Learner contains two options for dividing data into training and testing: cross-validation which randomly divides data into desired number of groups, and holdout which randomly divides data into two groups. As the aim of this study is to build user-independent models, leave-one-subject-out -method needs to be used in the final model training process, and therefore, neither of the approaches provided by Regression Learner are valid for the purpose. Moreover, both approaches lead to over-trained models as same persons data can be in training and testing sets. However, though Regression Learner cannot be used to calculate the final outcomes of this paper, it can be used to compare the performance of different regression algorithms when predicting the amount of stress, and help to select the right regression algorithm to calculate the final outcomes of this article.

In the end, 13 regression algorithms were compared by training regression models using 5-fold cross-validation, see Table 1. As a result of this comparison, it was decided to use Bagged tree based ensemble model in the experiments as the root mean square error (RMSE) is the lowest using it.

## 4. Experiments

Experiments were made using classification and regression models. Moreover, user-independent and personal models were compared. The accuracies presented in this section are balanced accuracies, which is calculated by separately calculating accuracy for both classes and the reported balances accuracy is mean from these. Balanced accuracy was selected as the performance metric as it is not as vulnerable to unbalanced data as accuracy.

### 4.1. User-Independent Stress Detection

To compare regression and classification models, binary Random Forest classifier was trained and results of it were compared to Bagged Tree based ensemble regression model. Both were trained using leave-one-subject-out -method, meaning that in turn one person’s data were used for testing and others data for training. For classification model, target values were transformed as 1 and 0, so that data from baseline are labelled as 0 and data from driving as 1. However, regression model was trained using continuous target values. Moreover, as the outputs of the regression model are continuous values, they were transformed as binary by finding an optimal threshold for each person to divide outputs as stress and non-stress that maximizes the accuracy. This was done by analyzing the obtained continuous prediction values study subject-wise, and such stress level value was searched which divides the prediction values as stressed and non-stressed so that the obtained balanced accuracy rate is as high high as possible. This of course over-estimates the performance of regression model as the threshold is calculated based on personal data but still it shows the potential of regression model based stress detection.

The results of user-independent recognition rates using regression and classification algorithms and different sensor combinations are presented in Table 2. Using leave-one-subject-out cross-validation method, a own recognition model was trained for each study subject and the performance of the model was calculated using balanced accuracy, sensitivity and specificity. The values presented in Table 2 shows the mean balanced accuracies, sensitivities and specificities calculated over all nine study subjects, and standard deviation between the study subjects is presented in parenthesis.

Interestingly, according to Table 2 in both cases (regression and classification), the best recognition rates are obtained using only accelerometer features. This is not in line with previous stress detection studies, where it has been noted that features extracted biosignals are more useful for detecting stress than accelerometer features (see, for instance [19]). However, the reason for this is that, in this case, accelerometer-based models do not recognize stress at all; instead, they recognize the mode of transportation as baseline signal and stress signal are performed under different activity (sitting in non-moving car vs. driving a car). In fact, it is shown that these two activities can be recognized based on accelerometer data [20]. Therefore, as the aim of the study is to recognize stress, only models based on biosignals (=EDA, BVP, and ST) are worth studying.

Table 2 shows the potential of regression models. It outperforms classification algorithm with each sensor combination, and the highest recognition rates can be obtained using BVP and ST features. With this setting, the average balanced accuracy was 74.1% with classification model and 82.3% using regression model. In addition, sensitivity and specificity values are the highest using this combination. Confusion matrix for regression model for regression model using these features is shown in Table 3, where it can be seen that both classes are detected with reasonable accuracy. Moreover, according to Table 4 where user-independent accuracies are studied subject-wise using BVP and ST features, it can be noted that regression model performs better than classification model with all nine study subjects. Partly, the great performance of regression model is due to optimizing the threshold to classify outputs as stressed/non-stressed based on personal data but this is not the only reason for good performance of regression model. Another reason for it is that regression models gets more information as an input than classification model. In fact, classification models gets binary class labels as input while regression model’s inputs are continuous targets. This results show that stress detection benefits from continuous targets and is as a nature a regression problem instead of classification problem. This was expected, as the level of the stress of a person can be high or low or anything between these.

**Figure 1:**
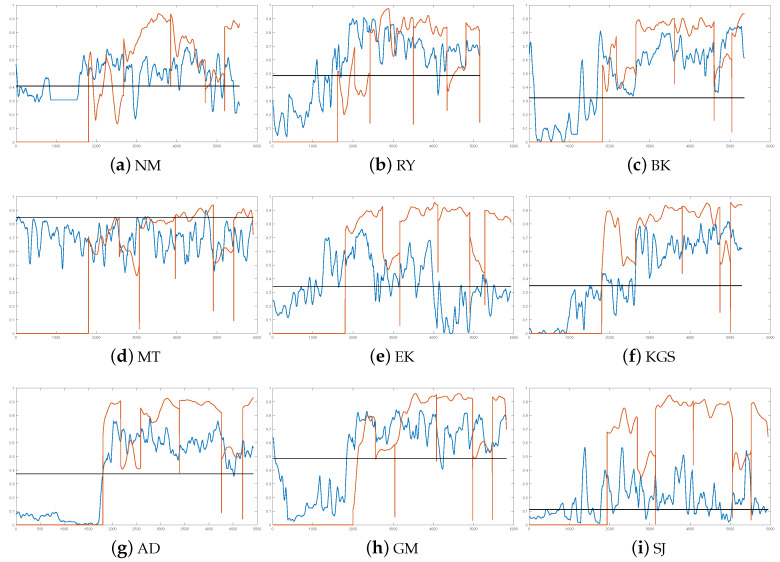
Predicted stress level (blue line) vs. subject estimated continuous target value for stress level (orange line). Personal threshold used to divide outputs as stressed and non-stressed is shown using black horizontal line.

While regression models outperform classification models in stress detection, the real benefit of regression models is that, unlike classification, they can be used to estimate the level of stress. Figure 1 shows how well regression models estimate the level of the stress compared to user reported stress level values. The figure shows that in most cases the regression model has approximately managed to predict the level of the stress. However, this approximation is not very accurate, and it is inconsistent. This suggests that the the trained regression models is very sensitive to small changes in feature values, and due to this, the value of predicted stress level can rapidly change. Moreover, the approximation does not work at all for some study subjects (see study subjects MT, Figure 1d and SJ, Figure 1i). In the case of MT, the model has not managed to recognize the non-stressed stage, and in the case of SJ, the recognition of stressed stage has caused problems. These exceptions suggest that stress does not cause similar reactions to every person. Moreover, Figure 1 shows the personal threshold used to divide regression model outputs as stressed and non-stressed (black horizontal line). In most cases, the value of this threshold is between 0.3 and 0.5. However, also in this case MT and SJ are exceptions. In the case of MT, the value of threshold is close to 1, and in the case of SJ, the value is close to 0. This underlines how special these the cases are compared to other seven study subjects.

Table 5 shows root mean square error (RMSE) of predicted and user reported values for each study subject. As the stress level of the baseline was not measured during the data gathering, it was assumed that during the baseline measurement the level of the stress was constant and zero. This of course is just a best guess, and therefore, cannot be considered as fact. In fact, also the predictions shown in Figure 1 suggests that the level of the stress is not constant during the baseline and neither it is zero. Due to this, RMSE values of Table 5 are calculated from two parts of the signal: the second column shows the RMSE value from the whole signal and the third column shows the RMSE calculated only from driving part of the signal as only it contained target values defined by the driver. For instance, in the case of subject MT, there is a big difference between these two. Moreover, the R-Squared value was calculated from each subject, and they are shown in Table 5. These also underline the fact that prediction does not work for each subject. In fact, R-Squared value is zero for three study subjects indicating that for these persons user-independent model is not able to predict any variation on response. In overall, the results of the Table 5 show the potential of predicting the level of the stress using regression models. However, it also shows that at this point of the research, it only works for some study subjects and the prediction can only give a rough estimation of the stress value, and it needs to be further studied how the exactness of the prediction could be improved.

The combination of BVP and ST features produced on average the highest recognition rates using user-independent regression model. However, this combination was not the best for each subject. Table 6 shows subject-wisely which sensor combination produces the best result. It can be seen that for most subjects, BVP + ST produces the best results but there is also exceptions. For instance for subject EK, a different selection of sensors improves the recognition rate over 20%-units (59.4 vs. 83.2, respectively). However, it has even bigger effect on standard deviation which is much smaller when features are selected subject-wise (17.0 vs. 11.7, respectively). Therefore, possibility to select features subject-wise, and this way personalize recognition model, could potentially have a significant positive effect to the recognition rates.

### 4.2. Personal Stress Detection

The dataset contains more that one data gathering session from three study subjects (three sessions from subject NM (named NM1, NM2 and NM3), two from RY (named RY1 and RY2) and GM (named GM1 and GM2). Therefore, these three study subjects can be used to experiment if the stress recognition models trained using personal data are more accurate than user-independent models, as it is suggested in some of the papers. The data gathering protocol was the same in each of these session, and on each session, the study subjects drove the same route. However, what makes each session unique is that traffic was different in each case as the study subjects drove on public roads.

User-dependent recognition rates for NM, RY and GM are shown in Table 7 using different sensor combinations, and classification and regression models. In each case, models are trained with data from one session and tested with data from another session from the same user. NM1, RY1 and GM1 were used for testing, as these were also used in previous section to train and test user-independent models. According to Table 4 using user-independent model and a combination of BVP and ST features, which based on the Table 2 provide the highest average accuracy, Subject NM’s stress can be detected with balanced accuracy of 64.3%/84.6% (classification vs. regression), RY’s 90.8%/93.7%, and GM’s 69.9%/95.0%. When these are compared to personal recognition rates based on the same features, it can be noted that personal training data do not have a big effect on the recognition rates. In fact, in the case of RY, the results based on personal model are much worse than the results based on user-independent model. Moreover, according to Table 7, the results using personal models are surprisingly bad no matter which sensor combination is used. There are some exceptions, though. RY’s results are really good when using BVP features, and GM’s stress can be recognized with high accuracy using a combination of BVP and ST features. However, the results from these users were already really good using user-independent model and usage of personal data do not have a big effect compared to those.

There can be several reasons for low personal recognition rates. For instance, in the case of data from subject GM, there seems to be problems with EDA signal, and thus, prediction using it always leads to really bad results. Another reason for low personal recognition rates can be that the size of the training data was not big enough, as in the case of personal model training data consist of measurement from one session and in user-independent case it consisted of measurements from eight sessions. In fact, when NM1 was trained using data from two personal data gathering sessions (NM2 and NM3), the results were much better than the one’s reported in Table 7, and over 98% balanced accuracy was obtained using BVP and ST features, see Table 8. However, also in this case it can be noted that the recognition rate is highly dependent on which data are used for training and which for validation. It can be noted that when NM2 and NM3 are used for training and NM3 for testing, the recognition rates are really bad no matter which sensor combination is used. This shows that there are a lot of variation between data gathering sessions, even if the the data are collected from the same person.

## 5. Discussion and Conclusions

In this article, regression and classification models were compared for stress detection. Both personal and user-independent models were experimented. The article was based on publicly open dataset [14], which unlike most of the other stress detection datasets, contained continuous target variables. The used classification model was Random Forest and the regression model was Bagged tree based ensemble.

The study shows that regression models are superior to classification models when it comes to user-independent stress detection, if continuous target values are available. The best results were obtained using a combination of BVP and ST features, and using these the average balanced accuracy was 74.1% with classification model and 82.3% using regression model. In fact, basically no matter which sensor combination was used, the results using regression models were better than the one’s obtained using classification models. This is because during the training process, regression model gets more information than classification model: regression model gets continuous targets as input while classification models inputs are discrete. Moreover, the results show that stress is not binary problem, a person can be highly stress, not stressed at all or anything between these two. Therefore, the results of this article show that prediction model should be trained so that all the available can be given as an input to it.

The main advantage of using regression models is that they can not only be used to recognize whether the person is stressed or not, they can also be used to predict the level of the stress. However, based on our experiments this prediction is not very accurate, and for some study subjects it does not work at all. In fact, the main goal of the future work is to study how the quality of this prediction could be improved. Due to this, different regression models needs to be compared to find out which is the most capable to predict the level of the stress. This also includes comparison of different quantitative indicators to measures the goodness of the prediction. Unfortunately, continuous target variables are rarely available for the datasets which is the reason why regression models are not often used for stress detection, and why stress detection based on classification models is an important topic to study also in the future.

Stress detection based on personal training data was also studied using data from three persons. The results obtained using personal models were surprisingly bad. The situation was better when more personal data were used for training but still there was a lot of variation in the recognition rates depending on how data gathering sessions were divided for training and testing. This shows that biosignals have a lot of variation not only between the study subjects but also between the session gathered from the same person. Therefore, personal recognition models should be able to constantly adapt to changes in human body. Due to this, for instance incremental learning-based method should be experimented [21]. On the other hand, it was shown that subject-wise feature selection for user-independent model can be a better approach than usage of personal training data to personalize and improve recognition models. It was noted that with subject-wise feature selection, the average detection rate improved 4%-units, and it was especially useful to reduce the variance in the recognition rates between the study subjects.

Moreover, it can be noted that the recognition rates obtained in this article are not as high as the one’s obtained in several other stress detection studies where the same features are used ([7,19]). The reason for this can be used data gathering protocol. In many other studies, the stress data are collected from contexts (giving public speech, performing difficult arithmetic tasks, etc.) that most likely are more stressful than driving a car. Therefore, the dataset used in this study does as big difference between relaxed and stressed state as other datasets making it more difficult to analyze. It is also possible that the type of the stress is different in these contexts. In fact, part of the future work is to make experiments using different datasets. In fact, to extend this study, these datasets should include data from new types of sensors (for instance electroencephalography (EEG)), to show that the proposed method is not dependent on the used sensor. The expected result is that the proposed method works similar to this study if appropriate features are extracted from the user sensor. In fact, extracted features needs to be selected sensor-wise, and though, the same features should not be extracted from each sensor. In addition, the experimented datasets should contain data not only from stress, but also from other affect states to show that the proposed method is not dependent on the studied affect state. The final aim is to detect multiple affect states from at same time, for example a person can be stressed and angry. This could be done for instance by training an own regression model for each affect state.

Lastly, it should be noted that when dealing data from stress and affect states which is labeled by the study subjects themselves, it should be noted that study subjects do not necessarily know what they feel [22]. Therefore, the target variables obtained from the study subjects can be unreliable. Due to this, while the preliminary results presented in this article are encouraging, more experiments with more datasets should be carried out to get a better understanding of how accurate the presented method actually is.

## Figures and Tables

**Table 1:** Comparison of regression models using 5-fold cross-validation.

Method	RMSE
Linear regression	0.23
Robust linear regression	0.24
Fine tree	0.05
Medium tree	0.06
Coarse tree	0.08
SVM with linear kernel	0.24
SVM with quadratic kernel	0.14
SVM with cubic kernel	0.7
SVM with fine Gaussian kernel	0.14
SVM with medium Gaussian kernel	0.8
SVM with coarse Gaussian kernel	0.21
Boosted tree based ensemble	0.16
Bagged tree based ensemble	0.03

**Table 2:** Average recognition results accuracies, sensitivities and specificities (standard deviation in parentheses) using regression and classification model and sensor combinations.

Regression			
Sensors	Balanced accuracy	Sensitivity	Specificity
ACC+EDA+ST+BVP	89.0 (13.3)	86.9 (22.7)	93.6 (5.8)
EDA+ST+BVP	75.0 (16.0)	71.6 (27.3)	90.9 (9.2)
EDA+BVP	75.1 (15.8)	73.5 (24.5)	88.3 (7.8)
BVP+ST	82.3 (17.0)	76.6 (23.8)	91.0 (6.1)
EDA+ST	72.3 (16.7)	70.9 (24.2)	84.0 (11.0)
EDA	73.5 (15.2)	72.0 (23.7)	87.5 (10.8)
BVP	78.5 (16.7)	72.1 (23.7)	89.0 (7.2)
ST	68.6 (11.8)	56.1 (19.3)	83.5 (9.0)
ACC	94.3 (5.5)	93.2 (7.0)	96.2 (3.4)
**Classification**			
Sensors	Balanced accuracy	Sensitivity	Specificity
ACC+EDA+ST+BVP	85.2 (14.2)	82.4 (22.9)	91.7 (9.4)
EDA+ST+BVP	65.4 (22.8)	61.6 (33.9)	76.7 (15.7)
EDA+BVP	69.3 (16.9)	57.9 (33.3)	78.7 (11.4)
BVP+ST	74.1 (16.7)	66.0 (33.5)	85.6 (10.9)
EDA+ST	64.6 (23.1)	54.1 (31.8)	73.2 (17.5)
EDA	64.5 (14.6)	55.8 (23.9)	78.1 (12.8)
BVP	70.4 (20.4)	64.0 (38.2)	82.9 (11.0)
ST	61.3 (14.9)	50.5 (27.6)	76.5 (12.0)
ACC	90.2 (10.1)	91.4 (9.2)	96.0 (6.4)

**Table 3:** Confusion matrix, when user-independent regression model based on BVP and ST features is used for stress detection.

True/Predicted	Non-Stressed	Stressed
**Non-stressed**	88.0%	12.0%
**Stressed**	26.6%	73.4%

**Table 4:** Subject-wise balanced accuracy using user-independent model and a combination of BVP and ST features.

Subject	Classification (%)	Regression (%)
NM	64.3	84.6
RY	90.8	93.7
BK	87.0	88.3
MT	49.0	50.5
EK	59.4	60.7
KGS	88.3	93.7
AD	96.3	99.7
GM	69.9	95.0
SJ	62.2	74.7
Mean	74.1 (STD 16.7)	82.3 (STD 17.0)

**Table 5:** Subject-wise RMSE and R-Squared using user-independent model and a combination of BVP and ST features.

Subject	RMSE Total	RMSE Stress	R-Squared Total
NM	0.31	0.26	0.09
RY	0.27	0.26	0.49
BK	0.24	0.18	0.60
MT	0.43	0.18	0
EK	0.46	0.52	0
KGS	0.25	0.28	0.60
AD	0.20	0.25	0.75
GM	0.22	0.21	0.64
SJ	0.48	0.60	0
Mean	0.32	0.31	0.35

**Table 6:** Best subject-wise recognition rates obtained using user-independent regression model, and which sensor combination was used to obtain this result.

Subject	Balanced Accuracy (%)	Sensor Combination
NM	84.6	BVP+ST
RY	93.7	BVP+ST
BK	91.1	EDA+BVP+ST
MT	64.3	EDA+BVP+ST
EK	83.2	BVP+EDA
KGS	93.7	BVP+ST
AD	99.7	BVP+ST
GM	95.0	BVP+ST
SJ	70.8	ST
Mean	86.3 (STD 11.7)	

**Table 7:** Recognition rates of users NM, RY, and GM using models trained using personal data.

Sensors	Valid/Train NM1/NM2 Classif./Regr.	Valid/Train NM1/NM3 Classif./Regr.	Valid/Train RY1/RY2 Classif./Regr.	Valid/Train GM1/GM2 Classif./Regr.
EDA+ST+BVP	69.3/70.1	49.2/68.0	50.0/50.0	50.0/ 71.4
EDA+BVP	69.9/79.5	50.0 /50.0	54.8/71.2	50.0/50.0
BVP+ST	69.3/88.0	50.8/67.9	50.0/65.0	83.7/91.6
EDA+ST	69.3/70.1	80.9/91.4	50.0/50.6	50.0/50.0
EDA	69.2/83.5	49.4/68.8	51.1/50.5	50.0/50.0
BVP	83.3/84.9	51.4/50.8	95.0/94.4	57.8/91.1
ST	69.3/70.1	69.3/69.4	50.0/84.7	50.0/78.7

**Table 8:** Cross-validation of three datasets collected from study subject NM.

Sensors	Valid/Train NM1/NM2 + NM3 Classif./Regr.	Valid/Train NM2/NM1 + NM3 Classif./Regr.	Valid/Train NM3/NM1 + NM2 Classif./Regr.
EDA+ST+BVP	74.4 /98.3	77.5/99.3	50.1/52.6
EDA+BVP	62.4/77.2	32.0 /50.0	51.0/52.4
BVP+ST	85.5/98.8	50.0/60.4	52.3/52.1
EDA+ST	69.3/85.6	80.6/99.4	48.2/52.6
EDA	63.8/80.1	32.9/68.3	49.6/51.0
BVP	74.5/78.7	64.8/87.4	51.9/50.9
ST	69.3/69.4	50.0/51.1	56.9/61.2
